# Supplementary material for: Considerations for Using Neuroblastoma Cell Lines to Examine the Roles of Iron and Ferroptosis in Neurodegeneration
Source: Cells. 2024 Sep 13;13(18):1541. doi: 10.3390/cells13181541 (PMC11430288; doi:10.3390/cells13181541)
Supplement: Supplementary file 1 [file cells-13-01541-s001.zip › cells-3186885-supplementary.pdf]

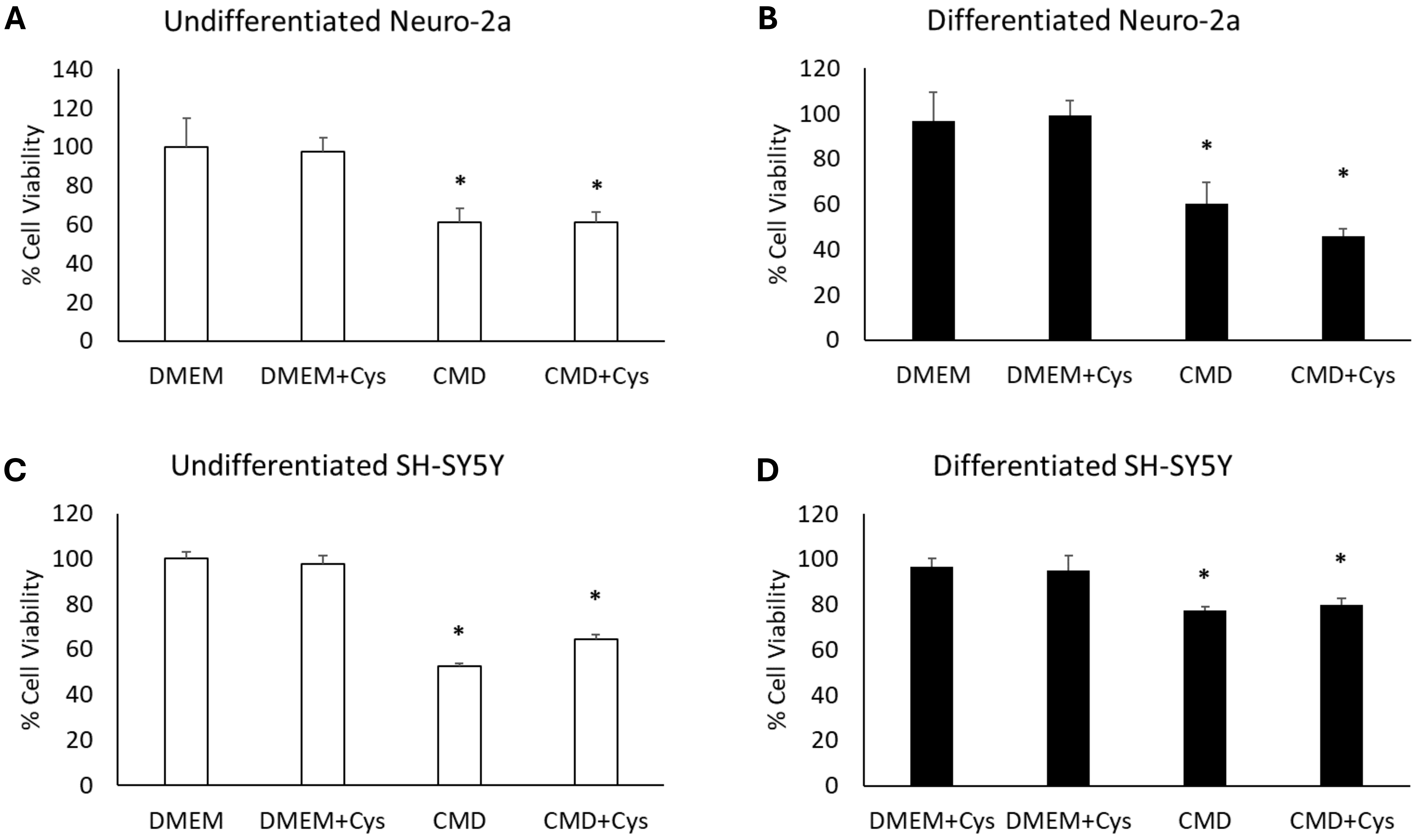

Supplemental Figure S1. Relative cell viability of (A) undifferentiated and (B) differentiated Neuro-2a cells following 24 hours of growth in either control DMEM or cysteine and methionine depleted DMEM (CMD). Relative cell viability (C) undifferentiated and (D) differentiated SH-SY5Y following 72 hours of growth in either control DMEM CMD. Supplementation with 200 μM cystine (+Cys) did not rescue cell growth in either cell line under control conditions.

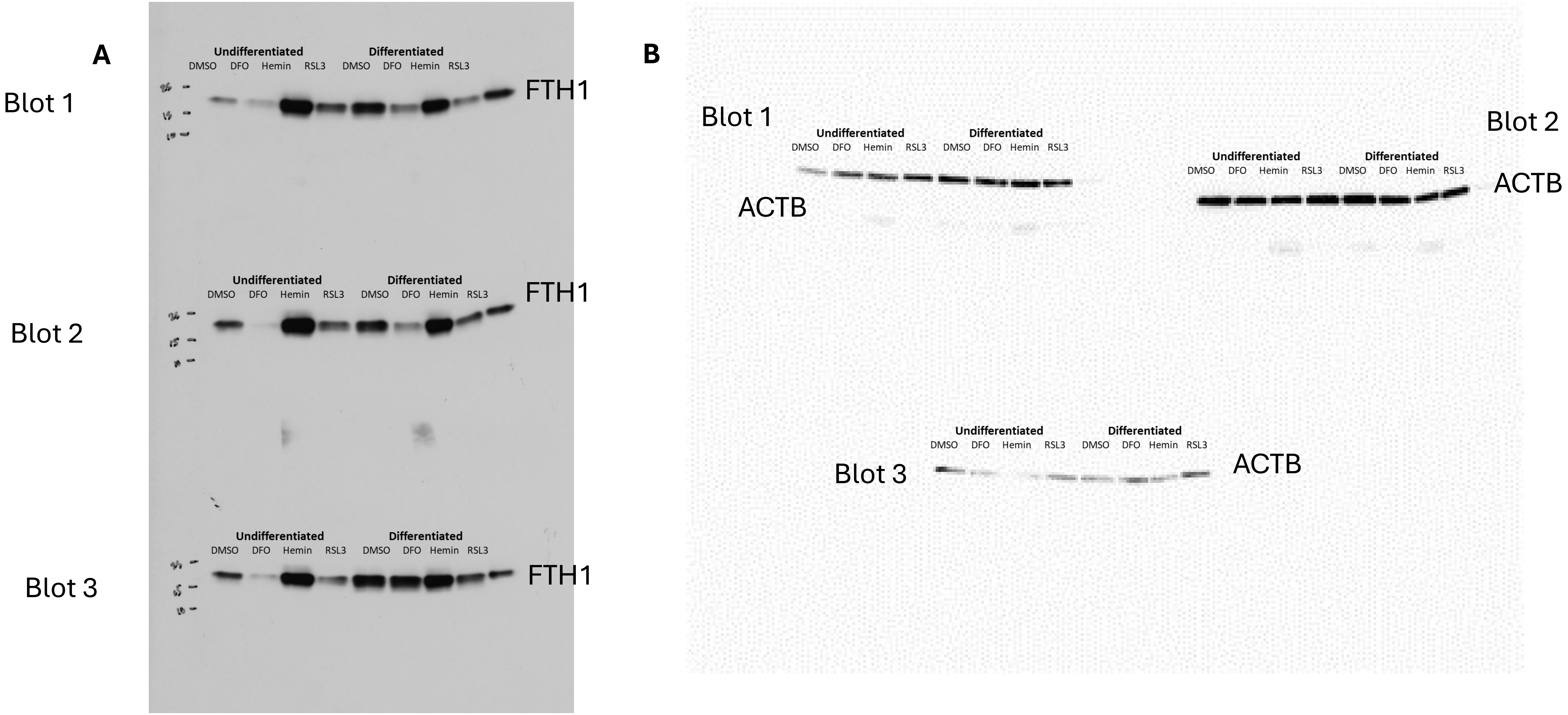

Supplemental Figure S2. (A) Uncropped images of SH-SY5Y ferritin protein expression in 3 replicate blots exposed to film and (B) corresponding images b-actin expression. All 3 blots were exposed on the same piece of film to assess ferritin expression. After which, blots were probed for b-actin and exposed to another single sheet of film to control for exposure times.

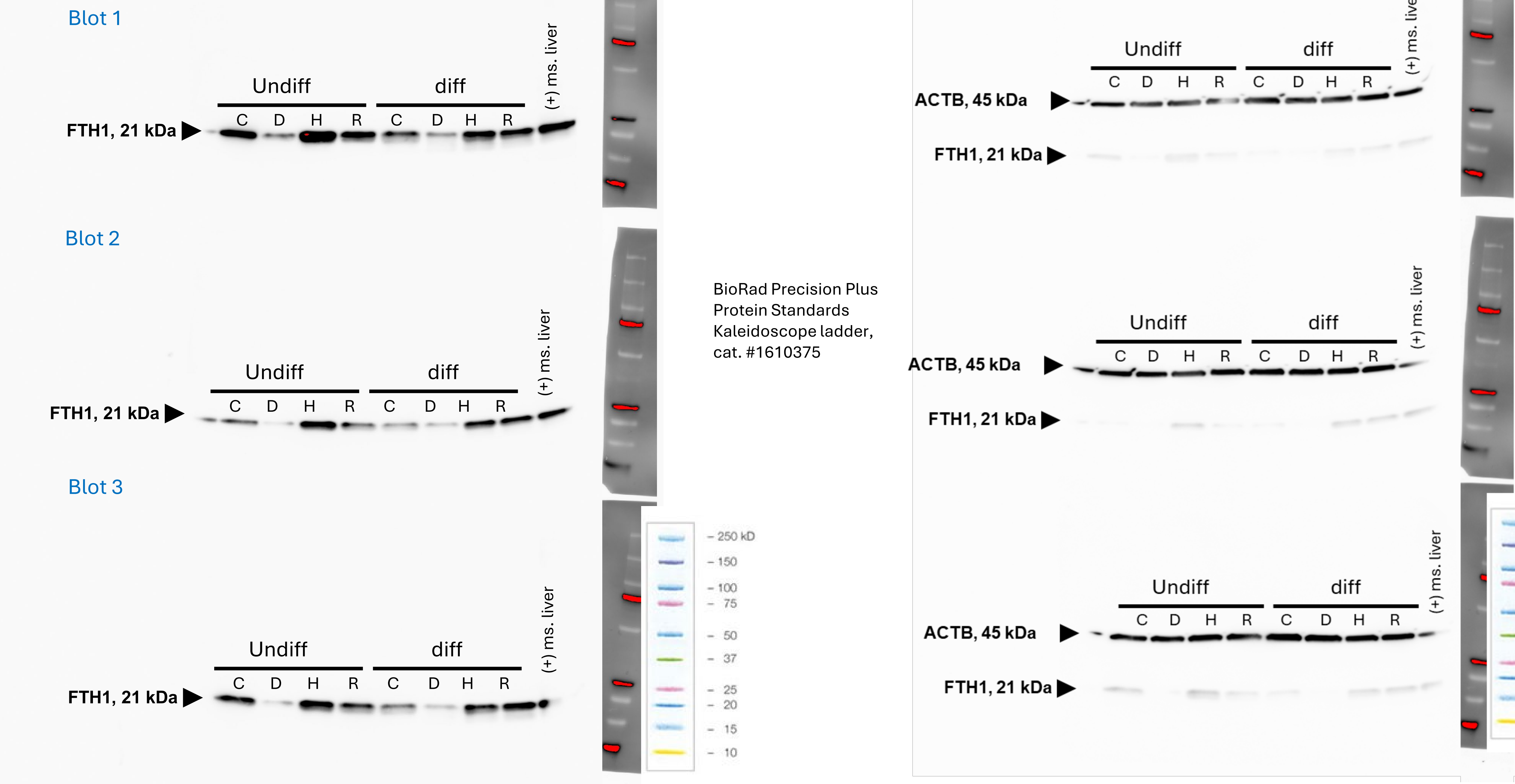

Supplemental Figure S3. (A) Uncropped images of Neuro-2a ferritin protein expression in 3 replicate blots exposed to film and (B) corresponding images b-actin expression. All 3 blots were exposed on the same piece of film to assess ferritin expression. After which, blots were probed for b-actin and exposed to another single sheet of film to control for exposure times.

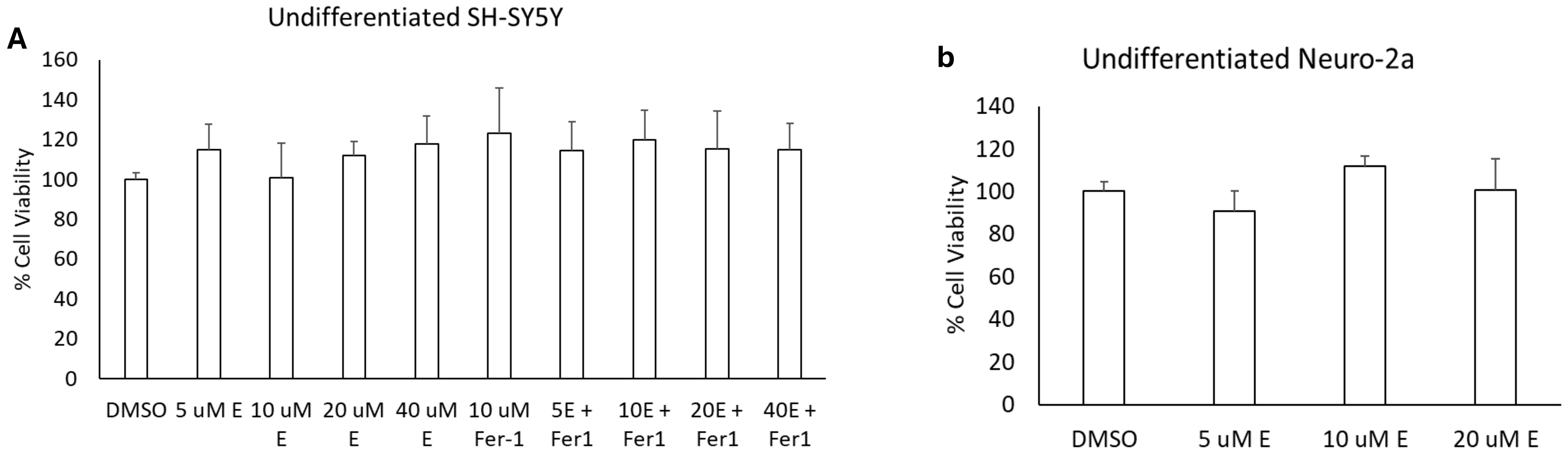

Supplemental Figure S4. Relative cell viability of (**A**) undifferentiated SH-SY5Y and (**B**) Neuro-2a cells following 48 hours of treatment with the indicated doses of erastin (E). SH-SY5Y cells were also co-treated with the ferroptosis inhibitor ferrostatin-1 (Fer1), but it did not have a positive or negative effect on cell viability either.

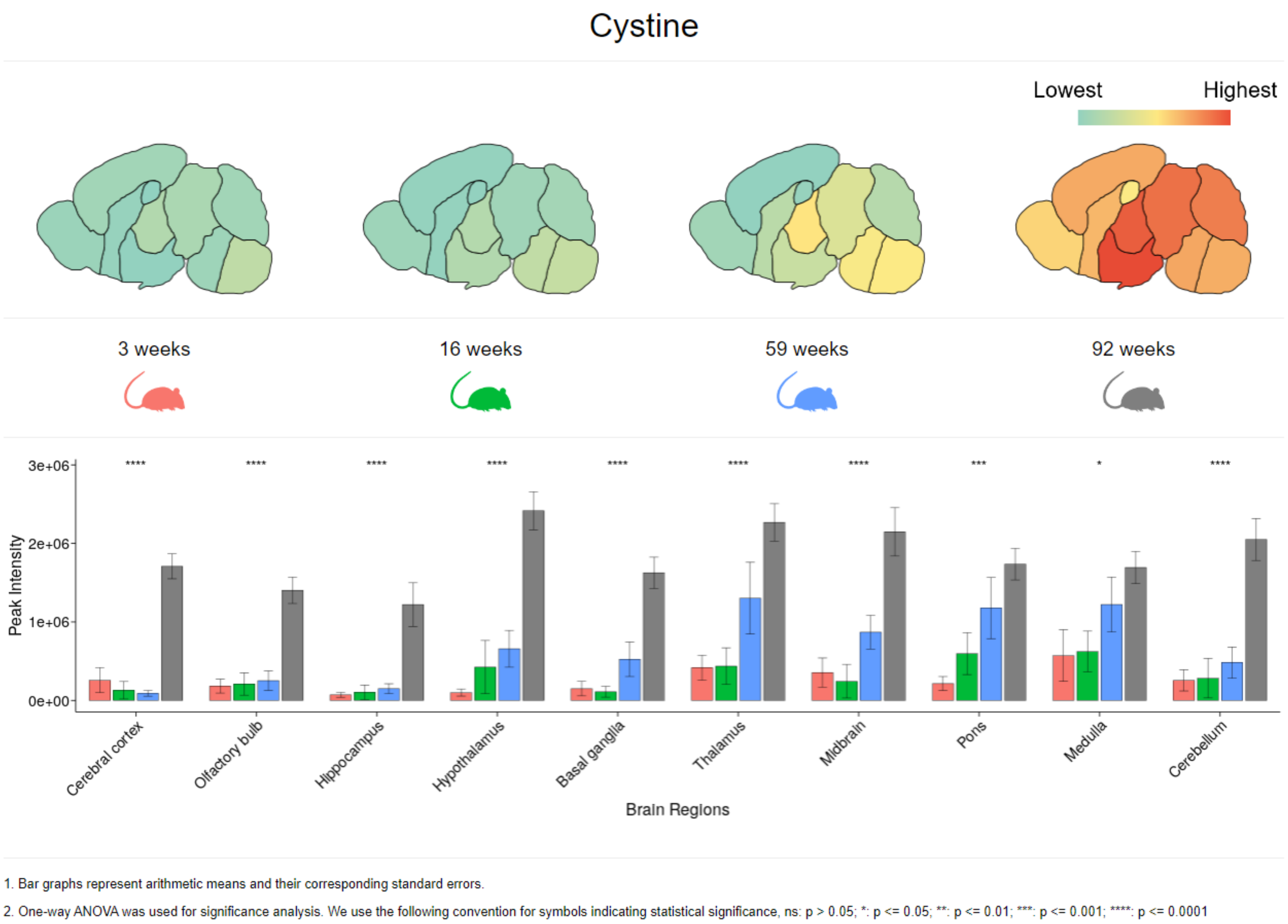

Supplemental Figure S5. Cystine levels in 10 different brain regions from aged mice derived from: https://mouse.atlas.metabolomics.us/
